# Claims-based measures of continuity of care have non-linear associations with health: data linkage study

**DOI:** 10.23889/ijpds.v3i1.463

**Published:** 2018-12-04

**Authors:** Bich Tran, Michael Falster, Louisa Jorm

**Affiliations:** 1 Centre for Big Data Research in Health, UNSW Australia Sydney NSW 2052, Australia

## Abstract

**Background:**

Continuity of care (CoC) is considered a central element of good primary care and is often measured using medical claims data. Possible values of CoC depend on the number of claims which is related to health status. This study investigated the relationships between CoC and health status and risk of emergency hospitalisation.

**Methods:**

Health insurance claims for consultations with general practitioners (GPs) in the 24 months following entry to the 45 and Up Study were used to calculate usual provider continuity (UPC) and the Continuity of Care Index (CoC Index). Relationships of CoC with number of claims, self-rated health and emergency hospitalisation were investigated using descriptive statistics and logistic regression models.

**Results:**

Both measures of CoC were strongly related to number of claims and to measures of health status, which were in turn highly associated. Multivariable logistic regression models showed a weak positive relationship between CoC and odds of emergency hospitalisation for those with CoC less than 1, while individuals with perfect CoC had significantly lower odds of hospitalisation compared to all other categories of CoC. However, analyses stratified by, or adjusting for, number of claims showed no clear associations between CoC and risk of hospitalisation.

**Conclusions:**

The pattern of association between CoC categories and emergency hospitalisation was non-linear and was confounded by the effect of number of claims. Future studies should apply caution in using claims-based measures of CoC as a continuous variable or employing an arbitrary cut-point, and should adjust for number of claims.

## Background

Continuity of care (CoC) is recognised as a central element of good primary care, and the potential role of CoC in driving improved health outcomes is highly topical. Broadly, CoC can be considered as continued contact between a patient and their healthcare provider (1), and achieving high CoC is a major driver behind international health care reforms such as Patient Centered Medical Homes in the US (2) and Health Care Homes in Australia (3).

Measuring CoC poses challenges. There is lack of consensus about defining and measuring CoC, and previous studies have defined CoC according to multiple dimensions including informational, longitudinal, geographic, interdisciplinary and interpersonal CoC (4, 5). Among these, interpersonal CoC, which refers to a relationship between a patient and one or more providers that extends beyond specific episodes of illness, is the most frequent focus of research (6). Interpersonal CoC can be measured using health insurance claims for visits to specific health care providers (7). There are at least 17 different claims-based indices for measuring CoC (6), which can be generally grouped into three major categories: density of patient’s visits, dispersion of care providers, and sequence of visits to providers (8). Many of these measures have similar methods of construction and are highly correlated (9, 10).

Empirical evidence suggests a beneficial effect of claims-based CoC (11), mostly using density or dispersion measures, on health service utilisation (12-16), medication adherence (17), unnecessary medical procedures (7), cost (18) and health outcomes including mortality (12, 19). However, little attention has been paid to further interrogating these associations, with most previous research assuming the linear effect of CoC on outcomes by treating this as a continuous (7, 15) or a dichotomous variable (13, 16, 18). Further, the possible values of CoC are related to the number of claims used in their calculation, and number of claims is in turn highly related to health status. In this study, we examined the relationship between CoC and emergency hospitalisation using more comprehensive categories of CoC, to test the pattern of the association and to investigate potential effect of number of claims.

## Methods

### Study design

This analysis was part of the Assessing Preventable Hospitalisation InDicators (APHID) study. The APHID study protocol has been published elsewhere (20). Briefly, APHID uses linked survey and administrative data for participants in the Sax Institute’s 45 and Up Study, a prospective cohort of over 267,000 men and women aged 45+ years and resident in New South Wales (NSW), Australia (21). Participants were randomly recruited between Jan 2006 to Dec 2009 from the database of the national health insurance scheme (Medicare Australia). Participants entered the study by completing a mailed self-administered questionnaire at baseline (22) and providing consent for long-term follow-up, including linkage to a range of administrative health data sets. People residing in non-urban areas and those aged 80 years and over were oversampled. The overall response rate for the 45 and Up Study was 18% and the study included about 10% of the NSW population aged 45 and over.

Ethics approval for the 45 and Up Study was granted by the University of New South Wales Human Research Ethics Committee, and approval for the APHID study was granted by the NSW Population and Health Services Research, Aboriginal Health and Medical Research Council and University of Western Sydney Research Ethics Committees.

### Data sources

Participants’ socio-demographic and health characteristics were derived from a self-reported questionnaire administered at study entry, apart from the measure of remoteness of residence, which was assigned according to the mean score of the Accessibility Remoteness Index of Australia Plus (ARIA+) for the Postal Area of the participant’s address (SLA) (23). Socio-demographic variables (**Table 1**) included participants’ age, sex, educational level, language spoken at home, marital status, annual household income, number of health behaviours (24), and private health insurance status.

Measures of health status included self-reported health status, number of co-morbidities, level of functional limitation (using the Medical Outcomes Study physical functioning scale) (25) and level of psychological distress (using the K10 Scale) (26).

## Data linkage and exclusion criteria

Almost all GP services in Australia are provided privately under a fee-for-service basis with a rebate contributed by the national health insurance scheme, Medicare Australia, at a level set by the Medicare Benefits Schedule (MBS). MBS claims for general practitioner (GP) consultations were obtained from the Department of Human Services, which administers Medicare.

Emergency hospitalisations were ascertained from the NSW Admitted Patient Data Collection (APDC), which captures all separations (i.e. hospital episodes or spells) from public and private sector hospitals in NSW.

All-cause mortality was ascertained from death registrations in the Registry of Births, Deaths and Marriages (RBDM) mortality data.

Linkage of 45 and Up Study cohort data to APDC and RBDM was performed by the Centre for Health Record Linkage (CHeReL) using probabilistic record linkage methods (ChoiceMaker, ChoiceMaker Technologies Inc.). CHeReL quality assurance data showed false positive and negative rates of 0.4% and less than 0.1%, respectively. Linkage of Medicare data was performed by the Sax Institute, using a unique scrambled Medicare identifier generated for the 45 and Up study.

Participants who died during the measurement period were excluded because they did not have a full data available, as were holders of a Department of Veterans’ Affairs (DVA) healthcare card, because MBS claims data do not capture all services provided to these cardholders.

## Measures of CoC

Insurance claims processed by Medicare Australia for GP consultations in the 24-month period following study entry were used to calculate measures of CoC. Claims were obtained from MBS data using item groups “A1” and “A2” which represent non-referred consultations with GPs in-hours at clinics, home visits and residential aged care facilities, but not hospitals. In Australia, general practitioners (GPs) serve as “gatekeepers” to specialist care and the rest of the health care system (27) and therefore claims for GP services are appropriately used to calculate CoC.

Only participants who had at least 4 GP claims were included, in consistent with previous practice (15, 28, 29). A separate analysis in those with at least 2 GP claims showed similar results (data not shown). Patients with only one claim by definition have perfect continuity, while minor changes in patterns of visits can have large impacts on CoC for patients with small numbers of claims (8). Participants with more than one claim per day on average were also excluded, because this number of claims was implausible, and may have resulted from errors in data linkage or erroneous claims.

Measures of CoC were calculated using the most two common metrics: the usual provider continuity (UPC) and the Bice-Boxerman CoC Index (CoC Index). Usual provider continuity (UPC) (30), measuring the proportion of patient’s claims to the most common provider, was calculated using the formula UPC = $\frac{\max(n_{i})}{N}$ while the Bice-Boxerman CoC Index (CoC Index) (31), measuring the dispersion of patient’s claims across all providers, was calculated using the formula CoC Index = $\frac{(\sum_{i=1}^{k}n_{i}^{2})-N}{N(N-1)}$, where *k* is the number of providers and *n^i^* is the number claims to provider *i* and *N* is the total number of claims to all providers.

## Emergency hospitalisation

Any emergency hospital admission in the 12 months following the measurement period for CoC was used as the study outcome. Number of emergency hospitalisations in the 12 months prior to study entry was also used as a covariate in the analysis.

## Statistical analyses

The distribution of CoC measures was examined, both overall and stratified by quartile of the total number of GP claims (Q1=4-7 claims; Q2=8-12; Q3=13-19 and Q4=20+). Values of CoC were then categorised into 4 groups: <0.5; 0.5-0.74; 0.75-0.99 and 1.0, to characterise both the breadth of scores as well as the traditional cut-point for ‘high’ CoC (0.75 and above). Chi-squared or Wilcoxon-Mann-Whitney tests, where appropriate, were used to test differences in socio-demographic, health characteristics and health services utilisation, with the lowest CoC group being used as a reference. The relationships between number of claims and measures of health status (self-reported health status, number of comorbidities, levels of functional limitation and psychological distress) were assessed using Chi-squared tests and ordinal logistic regression.

The association between CoCs and risk of emergency hospitalisation was estimated using multivariable logistic regression, and presented as odds ratios with 95% confidence intervals (CI). Negative binomial regression was also used to estimate the effect of CoC on number of emergency hospitalisations and the patterns of association was similar (data not shown). We included participants’ socio-demographic and health characteristics as potential confounders. To examine the effect of number of claims, quartile was further added as a covariate; and separate models, stratified by quartiles, were constructed.

In order to investigate the effect of a possible increase in seeking health services relating to a deterioration in health, sensitivity analyses were performed for a group of participants without hospitalisation in the measurement period. All analyses were performed in SAS version 9.4 (SAS Institute, Inc, Cary, NC) (32) and figures were produced using R (version 3.3.2) (33).

## Results

Among a total of 266,950 participants in the 45 and Up Study, 44,007 (16%) were excluded (8,082 who died; 4,216 who held a DVA healthcare card; 11 who had an average of >1 GP claim per day; and 31,698 who had less than 4 GP claims), leaving a total of 222,943 eligible participants for analysis. Among those with less than 4 GP claims, there was a higher proportion of people who were male, younger, spoke English at home, had higher levels of education, lived in outer regional areas, and were healthier, compared to those with 4 or more GP claims (data not shown). The average age of eligible participants was 63 years (standard deviation 11 years) and 55% were female. There was an average of 15 GP claims per participant (median 12, interquartile range [IQR] 8-19) during the 24-month measurement period, with services being provided by an average of 3 different GPs (median 2, IQR 1-4).

Almost half (49%) of participants had a UPC ≥ 0.75 (33% with UPC 0.75-0.99; 16% with UPC 1.0), whereas 32% had UPC 0.50-0.74 and 19% had UPC <0.50. There was a non-normal distribution of UPC scores both for all participants and within each quartile of number of GP claims (**Figure 1A**), with a spike at perfect continuity (UPC 1.0). Participants in the lowest quartile of number of claims (4-7 claims) had a limited set of UPC scores, reflecting those mathematically possible for each given number of claims.

Baseline health characteristics varied across different categories of UPC (**Figure 2A**), with an inverse relationship between UPC and self-rated health, number of comorbidities and levels of functional limitation for those with UPC less than 1 (**Supplementary Table 1**). However, individuals with perfect UPC (UPC 1.0) were more likely to be healthier than those with UPC 0.75-0.99. There was a notable difference in the number of GP claims between participants with UPC 0.75-0.99 and those with perfect continuity, with almost one-third (32%) of participants in the former category having ≥ 20 GP claims, whereas only 15% of participants in the latter category had this many claims (**Figure 2, Supplementary Table 1**).

There was an inverse relationship between number of claims and self-reported health status, number of co-morbidities, functional limitation and psychological distress, such that greater numbers of claims were significantly associated with poorer health status across all of these measures (all P values <0.0001) (**Supplementary Tables 2 and 3**).

In the 12 months following the CoC measurement period, 20,361 (9%) participants had at least one emergency hospitalisation. Compared to those in the lowest UPC category (UPC <0.5), participants with UPC of 0.5-0.74 (fully adjusted OR=0.98, 95% CI 0.94-1.03) and 0.75-0.99 (fully adjusted OR=1.03, 95% CI 0.99-1.08) had similar odds of hospitalisation (**Table 1**). However, those with UPC of 1.0 (i.e. perfect CoC) had 15% significantly lower odds of hospitalisation (fully adjusted OR=0.85, 95% CI 0.80-0.90) (**Table 1**).

In models stratified according to quartile of number of GP claims (4-7 GP claims, 8-12, 13-19, 20+), lower odds of hospitalisation for participants with perfect continuity was no longer observed (**Table 1**). Similarly, addition of the quartiles of number of claims as a covariate to the fully adjusted models attenuated the associations between CoC and hospitalisation, with no effect estimates attaining significance (**Supplementary table 4**).

Repeated analyses using Bice-Boxerman CoC Index (CoC Index) showed that 46% of participants had CoC Index <0.5, while CoC categories 0.5-0.74, 0.75-0.99 and 1.0 (perfect continuity) each included 14% of participants. Associations between categories of CoC Index, socio-demographic and health characteristics, and odds of emergency hospitalisation were similar to those observed for UPC (**Figures1B and 2B, Supplementary Tables 4 and 5, Table 1**). However, participants with CoC Index 0.75-0.99 had significantly elevated odds of hospitalisation (fully adjusted OR=1.11, 95% CI 1.06-1.16) compared with those with CoC Index <0.5, while as for UPC, the odds of hospitalisation was lowest for participants with perfect continuity (fully adjusted OR 0.87, 95% CI 0.83-0.91).

Sensitivity analyses excluding individuals who had been hospitalised in the 24 months after study entry (period used for calculating CoC) (N=31,637, 14%) showed similar results for the associations between CoC and hospitalisation (data not shown).

## Discussion

This study demonstrated that measures of CoC calculated using health insurance claims for GP visits were related both to number of claims and measures of health status. For individuals with less than perfect continuity (UPC or CoC Index 1.0), the number of claims increased, and measures of physical health declined, with increasing CoC. However, individuals with perfect CoC had fewer claims, and better health, than participants with high but not perfect CoC (UPC or CoC Index 0.75-0.99). Similarly, the pattern of association between CoC categories and risk of emergency hospitalisation was non-linear. Among those with CoC less than 1.0, CoC had a weak positive association with hospitalisation; while those with perfect CoC had a significantly lower odds of hospitalisation compared to the lowest category. However, these associations disappeared once stratifying on or adjusting for the number of patient claims.

While numerous previous studies have shown associations between CoC and a wide range of health outcomes, the majority of these have dichotomised measures of CoC using arbitrary cut-points (e.g. CoC 0.75) (13, 16, 18), or score percentiles (12, 14), while others have used CoC as a continuous variable (7, 15). In this study, individuals with ‘high’ continuity (CoC ≥ 0.75) comprised both those with the highest (UPC or CoC Index 0.75-0.99) and lowest (UPC or CoC Index 1.0) odds of emergency hospitalisation. Very few studies have further investigated the patterns of distributional associations with CoC. One previous study separated ‘high’ from ‘perfect’ CoC, and showed that 35% of patients had perfect CoC, compared to 16% in the current study. This variability suggests a critical need for future research to report the distribution of CoC and to reflect on how this may influence the patterns of association.

Prior studies have usually treated measures of CoC as independent predictors for health outcomes (7, 12-14, 16, 18, 19, 34). This study demonstrated that there is a strong relationship between number of claims, measure of health status, and claims-based measures of CoC. Analyses stratified by number of claims showed no clear associations between CoC and hospitalisation risk. This observation was congruent with a recent study which found an inconsistent pattern of association between CoC and hospitalisation risk within strata of number of physician contacts (35). Similarly, there were no significant associations between CoC category and hospitalisation risk in models adjustment for number of claims, suggesting the association was confounded by the effect of number of claims.

The current analysis also showed an association between number of claims and self-rated health status, comorbidities and functional status. This indicates that the confounding effect of number of claims is likely to relate to its relationship with health. However, number of claims is also potentially influenced by personal care-seeking behaviour (36) and the accessibility of care, and the role of these other factors may vary between health systems, particularly those where affordability issues limit access to care. Previous studies have found stark differences in the pattern of primary care use between countries (37), and this has resulted in vastly different patterns of association between primary care and hospitalisation (38).

Most studies have accounted for patient health through adjusting for measures of comorbidity, as self-report variable or derived through medical claims data. While practical, such measures can often be limited by the capture of medical conditions within these data sources. For example, Australian Medicare claims data contain no information about patient diagnoses. Use of linked data (e.g. hospital records) may assist in identifying comorbidities, but only for individuals who have had the relevant health events. Our findings suggest that stratification or adjustment for the number of claims may be a more appropriate means to control for patient health, as it can be considered a proxy for both severity of illness or propensity to seek care (39), is available for all users of the primary care system, and also helps account for the mathematical characteristic that only certain values for CoC are possible with differing numbers of claims. While some studies have already adopted this approach (15, 39, 40), it not widely utilised.

Strengths of this study included the large size of the cohort, and linkage to a number of routinely collected databases that provide virtually complete capture utilisation of a wide range of health services (21). This avoids recall bias and bias due to loss to follow-up. A limitation of the study is the lack of clinical data on reasons for GP consultations, or their outcomes. The use of individual-level data from a self-reported questionnaire provided independent information on health status and a wide range of personal and health characteristics. Nevertheless, findings from this study may not be generalisable to other patient cohorts. It is possible that other claims-based measures of CoC may perform differently to those that we used. However, the UPC and CoC Index are the two most commonly used measures of density and dispersion of care (8), and share both mathematical properties and high levels of correlation with a variety of other claims-based measures of continuity (9).

Findings from this study suggest that future studies, regardless of their setting, should apply caution in using claims-based measures of CoC as a continuous variable or employing an arbitrary cut-point, and should consider stratifying by, or adjusting for, number of claims.

## Availability of data and material

The data that support the findings of this study are available only through the Sax Institute’s SURE (Secure Unified Research Environment) facility but restrictions apply to the availability of these data, which were used under license for the current study, and so are not publicly available. Data are however available from the authors upon reasonable request and with permission of the Sax Institute.

## Figure Legends

Figure 1 Distribution of continuity of care score among all participants and by quartile of number of GP claims, for (A) usual provider continuity (UPC), and (B) Bice-Boxerman Continuity of Care Index (CoC Index)

Figure 2. Distribution of health characteristics and number of GP claims by category of (A) usual provider continuity (UPC), and (B) Bice-Boxerman Continuity of Care Index (CoC Index)

**Figure 1: Distribution of continuity of care score among all participants and by quartile of number of GP claims, for (A) usual provider continuity (UPC), and (B) Bice-Boxerman Continuity of Care Index (CoC Index):**
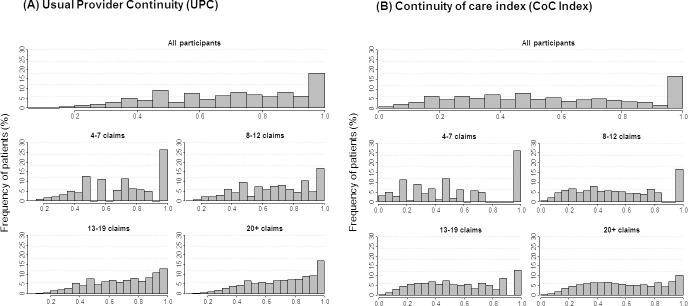


**Figure 2: Distribution of health characteristics and number of GP claims by category of (A) usual provider continuity (UPC), and (B) Bice-Boxerman Continuity of Care Index (CoC Index):**
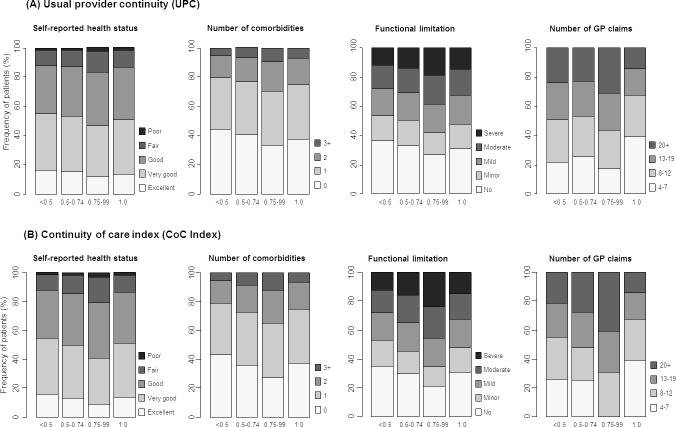


**Table 1: Association between continuity of care and emergency hospitalisation:** Fully adjusted model includes age, sex, language spoken at home, education, marital status, ARIA, private health insurance, household income, health behaviour score (non-smoking status, safe level of alcohol consumption (<14 drinks/week), at least 2.5 hours of intensity-weighted physical activity per week, and meeting daily dietary guidelines for fruit (2 serves) and vegetable (5 serves) consumption, number of comorbidities (on the basis of self-reported doctor-diagnosed heart disease, high blood pressure, stroke, diabetes, blood clot, asthma, Parkinson’s disease, and any cancer except skin cancer), functional limitation, psychological distress and emergency hospitalisations in the 12 months (yes/no) prior to study entry.

	Usual Provider Continuity (UPC)				Bice-Boxerman Continuity of Care Index (CoC Index)			
		
Category of continuity of care score	No emergency hospitalisations N (%)	1 or more emergency hospitalisations N (%)	Age and sex adjusted odds ratio (95% CI)	Fully adjusted odds ratio (95% CI)	No emergency hospitalisations N (%)	1 or more emergency hospitalisation, N (%)	Age and sex adjusted odds ratio (95% CI)	Fully adjusted odds ratio (95% CI)

**All data**								
<0.5	38,292 (18.9)	3,256 (16.0)	1	1	94,059 (46.4)	8,066 (39.6)	1	1
0.50-0.74	66,030 (32.6)	6,160 (30.3)	0.98 (0.94-1.03)	0.98 (0.94-1.03)	48,776 (24.1)	5,307 (26.0)	1.08 (1.04-1.13)	1.03 (0.99-1.07)
0.75-0.99	64,904 (32.0)	7,925 (38.9)	1.11 (1.06-1.16)	1.03 (0.99-1.08)	26,391 (13.0)	3,968 (19.5)	1.28 (1.23-1.33)	1.11 (1.06-1.16)
1	33,356 (16.5)	3,020 (14.8)	0.84 (0.80-0.89)	0.85 (0.80-0.90)	33,356 (16.5)	3,020 (14.8)	0.87 (0.84-0.91)	0.87 (0.83-0.91)

**Stratified by number of claims**								
**4-7 claims**								
<0.5	8743 (16.7)	298 (13.8)	1	1	25498 (48.8)	653 (44.0)	1	1
0.50-0.74	17681 (33.9)	696 (32.1)	1.08 (0.94-1.24)	1.07 (0.93-1.23)	13095 (25.1)	571 (26.3)	1.07 (0.96-1.19)	1.05 (0.94-1.16)
0.75-0.99	12169 (23.3)	530 (24.4)	1.13 (0.98-1.31)	1.10 (0.95-1.27)	0	0	.	.
1	13617 (26.1)	644 (29.7)	1.13 (0.98-1.30)	1.06 (0.92-1.22)	13617 (26.1)	644 (29.7)	1.07 (0.97-1.19)	1.01 (0.91-1.12)
**8-12 claims**								
<0.5	11508 (19.8)	590 (16.1)	1	1	28312 (48.7)	1577 (43.0)	1	1
0.50-0.74	18975 (32.6)	1135 (31.0)	1.06 (0.96-1.17)	1.05 (0.95-1.17)	11527 (19.8)	759 (20.7)	1.06 (0.97-1.16)	1.02 (0.93-1.12)
0.75-0.99	18038 (31.0)	1238 (33.8)	1.11 (1.00-1.23)	1.06 (0.96-1.18)	8682 (14.9)	627 (17.1)	1.10 (1.00-1.22)	1.05 (0.95-1.15)
1	9613 (16.5)	704 (19.2)	1.07 (0.95-1.20)	1.00 (0.89-1.13)	9613 (16.5)	704 (19.2)	1.03 (0.94-1.14)	0.97 (0.88-1.07)
**13-19 claims**								
<0.5	9783 (20.5)	807 (17.1)	1	1	21933 (46.0)	1890 (39.9)	1	1
0.50-0.74	15619 (32.8)	1405 (29.7)	0.98 (0.89-1.07)	0.94 (0.85-1.03)	12138 (25.5)	1234 (26.1)	1.05 (0.97-1.13)	1.00 (0.92-1.08)
0.75-0.99	16314 (34.2)	1820 (38.4)	1.10 (1.01-1.21)	1.02 (0.93-1.12)	7645 (16.0)	908 (19.2)	1.14 (1.05-1.25)	1.07 (0.98-1.16)
1	5955 (12.5)	701 (14.8)	1.05 (0.94-1.18)	0.96 (0.86-1.07)	5955 (12.5)	701 (14.8)	1.06 (0.97-1.17)	0.99 (0.90-1.09)
**20+ claims**								
<0.5	8258 (18.5)	1561 (15.9)	1	1	18316 (41.1)	3464 (37.2)	1	1
0.50-0.74	13755 (30.9)	2924 (29.9)	1.05 (0.98-1.13)	1.04 (0.97-1.12)	12016 (27.0)	2743 (28.0)	1.05 (1.00-1.11)	1.04 (0.98-1.10)
0.75-0.99	18383 (41.2)	4337 (44.3)	1.09 (1.02-1.16)	1.05 (0.98-1.12)	10064 (22.6)	2433 (24.8)	1.07 (1.01-1.14)	1.03 (0.97-1.09)
1	4171 (9.4)	971 (9.9)	1.03 (0.94-1.13)	1.02 (0.93-1.12)	4171 (9.3)	971 (9.9)	1.01 (0.93-1.09)	1.00 (0.92-1.09)

## List of abbreviations

APDC: the NSW Admitted Patient Data Collection; APHID: Assessing Preventable Hospitalisation InDicators; CoC Index: Bice-Boxerman continuity of care index; CHeReL: Centre for Health Record Linkage; CI: confidence interval; CoC: Continuity of care; DVA: Department of Veterans’ Affairs; GP: General Practitioner; MBS: Medical Benefits Schedule; RBDM: the Registry of Births, Deaths and Marriages; UPC: Usual Provider Index.
